# Becoming visible with limited resources: Non-profit journalists’ perspectives on search engine optimization

**DOI:** 10.1371/journal.pone.0322573

**Published:** 2025-04-29

**Authors:** Sebastian Schultheiß, Dirk Lewandowski

**Affiliations:** Department of Information, Media and Communication, Hamburg University of Applied Sciences, Hamburg, Germany; Akdeniz University, TÜRKIYE

## Abstract

This study examines the perspectives of non-profit online magazines regarding their online presence and the role that search engine optimization (SEO) plays in enhancing their visibility. We conducted semi-structured interviews with representatives from seven non-profit online magazines in Germany. Our findings indicate that visibility on the web is crucial for non-profits, even though they are not typically competing directly with commercial organizations. Non-profits seek visibility to achieve societal goals, such as educating their readership about fake news or promoting minority perspectives on political issues. Unlike for-profit organizations, economic factors are not their primary motivation. To reach their full potential audience, SEO is viewed as an important tool that all the surveyed magazines employ. However, due to limited resources, they cannot dedicate substantial effort to SEO and must treat it as a secondary task. The results of this study raise questions about how quality journalism from both for-profit and non-profit sources can reach the public in a balanced manner, as well as the responsibility search engine providers have in this context.

## Introduction

On the web, traffic is key to most content providers. The more traffic they get, the easier it is for them to sustain their businesses, e.g., through advertising or selling subscriptions. But what about non-profit providers? How important is visibility to them? And, given their limited resources, how do they achieve visibility to reach their target audience? In this article, we present results from qualitative interviews with non-profit news organizations in Germany, giving insight into strategies for gaining visibility with a specific focus on search engine optimization (SEO), a set of methods to increase the ranking of web pages in search engines.

Search engines have become an essential part of our daily lives [1]. They are used to search for a limitless range of topics, such as commerce, health, or society [2]. Search engines also play a crucial role in connecting news organizations with their audiences [3]. As shown by the Reuters Institute Digital News Report 2023 [4], which is based on samples representative of the online population of 46 countries, 30% of respondents cite social media as their main way of finding out about news online, followed by search engines (25%), and direct access to news websites (22%), the latter of which is declining. With a market share of 87% in the U.S. [5] and 90% in the EU [6], the mediating role of search engines is primarily fulfilled by Google. Among their users, search engines, notably Google, enjoy high trust and are regarded as neutral content providers, as surveys (e.g., [7,8]) and user studies (e.g., [9–11]) have shown. When looking for general news and information, people trust search engines (63%) more than traditional media (59%), owned media (47%), and social media (41%), as shown by the Edelman Trust Barometer 2023, an annual online survey with samples representative of the general population of 28 countries [12].

Search engines play a vital role not only as mediators of information but also as key traffic sources for media organizations [4]. In addition to the option of placing ads on search engine result pages through paid search marketing (PSM, see [13]), which may include subscription-related advertisements, search engine optimization (SEO) is of significant importance for both for-profit and non-profit media organizations.

SEO is utilized to enhance the ranking of a web page within the unpaid, so-called organic search results [2]. The importance of SEO for journalistic content stems from how users search for information online, for example, regarding current political issues or other socially relevant topics. When users conduct such searches, they are likely to encounter journalistic articles in the organic search results [14]. Their selection decisions heavily rely on the position of these results (e.g., [10,11]); hence, they tend to favor optimized (SEO) results. Once a user selects a search result, they may encounter advertisements on the journalistic websites, which increases the revenue generated from the traffic and makes organic traffic a crucial revenue source for profit-oriented news organizations [15–17]. The ubiquity of SEO has created interdependencies between the SEO industry and content producers [18], stabilizing the algorithmic ideology of search engine providers. Both content producers and users form “alliances with search engines to reach their own goals” [19].

In contrast, non-profit media organizations may be less dependent on traffic for financial reasons, as they receive funding from foundations, donations, or alternative revenue streams [20]. However, securing a prominent position in search results is essential for non-profits to create medium- to long-term societal impact through content like investigative reporting, health topics, or social justice issues [21]. This is because prominently placed search results have the potential to influence users’ opinions [22] and decisions on health, political, legal, or financial issues [23–26].

Overall, while both for-profit and non-profit media organizations depend on traffic to some degree, their starting points and strategies for achieving this traffic differ.

In its ideal form, the Web would always provide users with the content best suited to their needs and give a voice to less financially strong and less powerful providers such as non-profit media organizations [27]. Yet the ubiquity of SEO suggests that visibility on the Web can only be adequately achieved with SEO measures, which require resources. This could potentially give for-profit media companies an advantage over non-profit organizations [28]. For the latter, the resource demands could make SEO efforts more difficult to achieve, limiting the visibility of their web content and thus jeopardizing the mission to inform the public about socially relevant issues. This raises the question of how non-profit online magazines perceive the challenge of online visibility and how they view SEO measures to increase visibility. This paper investigates these questions through interviews with representatives of these organizations.

The rest of the paper is structured as follows. We present a literature review that provides an overview of for-profit online journalism and the importance of web visibility before discussing SEO as a resource issue. We then derive the research questions from the literature review and present the methods of the semi-structured interviews. Finally, we discuss the results, draw conclusions, and suggest avenues for future research.

## Literature review

### For-profit online journalism and the need for visibility on the Web

For-profit organizations dominate journalism compared to other funding models, such as non-profits, as shown by Nielsen et al. [29] for the independent news media sector. For-profit media organizations rely on two ways in which people pay for media content. One is by paying money, and the other is by investing time and attention to the content, which media organizations monetize through advertising [30]. Both financing methods are supported by search engine marketing (SEM), which distinguishes between paid search marketing (PSM) and search engine optimization (SEO).

PSM is part of search engine marketing and “is operated by search engines in the form of sponsored or paid results, where an advertisement is displayed in a pre-specified region of a search result page along with web search results” [31]. Compared to other fields of digital advertising, paid search marketing is the largest market. Spendings for search advertising reached $262 billion worldwide in 2023, followed by social media ($195 billion), video ($177 billion) and banner advertising ($162 billion), according to estimates by Statista Market Insights [32]. Apparently, the pandemic had a noticeable effect on search ad spending. Compared to the other advertising fields, search advertising saw the largest increase in growth rate, jumping from 11% in 2020 to 38% in 2021. Moreover, PSM is the foundation of Google’s business model. In 2023, Google’s parent company, Alphabet, generated almost $307 billion in revenue. Of that, $238 billion — about 77% — came from Google’s ads business [33].

Search engine optimization (SEO) is also part of SEM and places its focus on the unpaid organic results. SEO is “understood to mean all measures suitable for improving the position of Web pages in search engine rankings. These measures range from simple technical steps that help make documents indexable for search engines to complex manipulations of the linking structure of pages that refer to the documents to be optimized” [2]. As Schultheiß et al. [34] found for health-related web content using an SEO classification tool, optimized pages rank significantly higher than non-optimized pages. Furthermore, SEO is a steadily growing industry [35]. For 2021, total sales of 50 billion dollars had been forecast for the global SEO services market, although this estimate has been affected by COVID-19. The market is expected to recover from the impact of the pandemic and grow to 103 billion dollars in 2025 [36].

On the one hand, media organizations use sponsored results (i.e., paid search marketing) to encourage potential readers to pay money for their content. Search engine users often come across sponsored results from media organizations when entering queries intended to access a particular website. Such queries are so-called *navigational* queries [37]. For example, when searching on Google for *The Wall Street Journal* or other publishers, sponsored results promoting subscriptions appear on the search engine result page (SERP).

On the other hand, when searching with an *informational* intent, such as current political topics [37], users come across journalistic articles in the (unpaid) organic search result section. When selected, users will likely encounter the second financing method described above – advertising on journalistic websites next to editorial content. Thus, media organizations monetize their readers’ attention by selling it to advertisers [30]. Ad revenue is directly related to traffic [15,16] and is needed to maintain and improve the quality of the content [30]. Consequently, the business model of for-profit media organizations substantially depends on the traffic on their website. A look at the page impressions of news and media websites gives an idea of the importance of traffic obtained via search engines. For example, the page impressions coming from organic search for selected popular news and media websites range between 27% (wsj.com), 36% (cnn.com), 36% (bbc.com), and 43% (theguardian.com), as provided by data from Similarweb.com [38]. Consequently, for-profit media companies are keen to increase traffic and the associated advertising revenue. They do so with the help of SEO measures. SEO is utilized by most content providers, as reflected in analyses of web data. Using an SEO classification tool, Lewandowski et al. [39] found that the majority of pages found in Google’s top 130 ranking positions make use of SEO measures. SEO is also an omnipresent topic in newsrooms, as interviews with journalists show. Journalists describe SEO as crucial for the visibility of journalistic content on the Web [40,41]. Accordingly, for online journalists, SEO training is considered very important, as well as being up-to-date regarding current SEO developments [42].

The widespread use of SEO in newsrooms has also sparked discussions among journalists about its advantages and disadvantages. Looking at the benefits of SEO for journalism, SEO can be regarded as a value-neutral way to generate visibility for journalistic content on the Web [43]. As illustrated by the example of the BBC, implementing SEO measures significantly increased the number of visits to the website [44]. In addition, SEO can be a way for less popular sources to gain visibility with their content in Google News results, usually dominated by popular sources such as *The Guardian*, as Evans et al. [45] noted for the UK. When used moderately, journalists do not expect the quality of journalistic content to suffer from SEO measures, as statements from Spanish journalists indicate [40]. However, the close relationship between SEO and online journalism raises concerns about journalistic freedom, as highlighted by interviews with journalists from Indonesia [46], Great Britain [47], Greece [48], Germany [41], and Spain [40]. SEO can potentially jeopardize journalistic ethics [46], as “SEO is applying pressure to journalistic standards not in the perceived interest of the reader or because of publication constraints (as once may have been the case), but in the interests of a third-party commercial arbiter in online distribution: Google” [47]. This pressure directly impacts journalistic practices, as demonstrated by Greek journalists interviewed by Giomelakis et al. [48]. All interviewees agreed that SEO significantly influences how journalists and media professionals select news stories, often favoring trending topics and breaking news. Furthermore, it affects the publishing practices of media outlets, including the placement and duration of specific articles online [48]. Consequently, journalists in Germany and Spain expressed concerns about content quality when SEO is prioritized excessively in journalistic practice [40,41].

### SEO as a matter of resources

To achieve visibility through SEO, media organizations and other content producers require personal or financial resources to implement these measures through in-house SEO experts or online marketing agencies. This might give commercially motivated (i.e., for-profit) content providers an advantage in ensuring their visibility within the prominently placed results [28] and poses challenges for non-profit organizations, as these are likely to have less capacity for marketing activities than for-profit organizations [49,50].

For defining non-profit organizations (NPOs), the International Classification of Non-profit Organizations (ICNPO) [51] can be applied. The ICNPO was developed in the 1990s by scholars working on the *Johns Hopkins Comparative Non-profit Sector Project* to “capture most succinctly the reality of the non-profit sector in the thirteen different countries,” including the U.S. and Germany [51]. The classification uses the major economic activity of an organization as the key to classification and groups the non-profit sector into twelve major activity groups. These groups include, among others, the sectors of *education and research* (e.g., universities), *health* (e.g., public hospitals), *law, advocacy, and politics* (e.g., political parties), and *culture and recreation* (e.g., online magazines).

Looking at the ICNPO field of *education and research*, studies show that university websites only insufficiently operate SEO [52,53], leading to a decrease in the visibility of the universities’ content [54]. Similar observations have also been made for the *health* sector. Through interviews, Mager [55] found that nonprofit health information providers, such as diabetes associations, are less likely to adapt their pages according to SEO criteria. These statements are confirmed by an analysis of health-related web pages by Schultheiß et al. [34], which found that public authority websites and other non-profit providers performed SEO less frequently than for-profit providers such as pharmaceutical companies. The ICNPO field of *law, advocacy, and politics* covers, among others, political parties. In an analysis of personal websites of candidates in the run-up to the 2021 German federal election, Hinz et al. [56] found that 93% of candidates’ personal websites use SEO measures. A study by Caroleo et al. [57] came to similar results looking at political topics related to the Italian general election 2022. The authors found that websites of political actors are among the sites that most frequently use SEO, along with news and company websites.

Non-profit online journalism belongs to the ICNPO field of “culture and recreation” and usually deals with topics that for-profit companies do not cover or at least do not cover to a large extent, such as investigative reporting, arts, education, or other niche topics [20,21]. To the authors’ knowledge, there is no prior research on how non-profit online magazines perceive and use SEO.

### Research questions

As a basis for the research questions, we briefly summarize the literature. Commercially motivated media organizations use search engine marketing to improve the visibility of their content [28]. Traffic boosted by SEO and the resulting advertising revenue play a significant role in ensuring economic survival [30]. However, resources are required to implement SEO measures, posing challenges for non-profit organizations. According to the studies cited above, non-profit content providers use SEO less frequently than profit-oriented providers in the health and education sectors [34,52–55]. This finding could be due to SEO being considered less necessary for non-profits yet leads to disadvantages in ranking non-profit content on search engine result pages. In this paper, we investigate the perspectives on SEO of non-profit online magazines using the following research questions (RQs):

RQ1: How important do non-profit online magazines consider the visibility of their content on the web?
RQ2: Do non-profit online magazines promote the visibility of their content on the web, and if so, how?
RQ3: What role do non-profit online magazines’ financial and human resources play in implementing online marketing measures?
RQ4: What experiences do non-profit online magazines have with SEO?
RQ5: What attitudes do non-profit online magazines have towards SEO?

## Method: semi-structured interviews

We conducted semi-structured interviews with representatives of non-profit online magazines. The interviews were approved in written form by the institutional review board (IRB) of Hamburg University of Applied Sciences, Germany, with approval number 2023-15.

### Sample

In this section, we describe the recruitment of the interviewees. When researching for non-profit online magazines relevant to participate in the interviews, we came across an alliance that aims to strengthen non-profit journalism in Germany (to ensure the anonymity of the interview partners, we have decided not to mention the alliance’s name). Their supporters include online magazines as well as foundations and networks. We first picked out the online magazines (*N* = 16) and checked their revenue data in the CRIF Company Profiles database, which we accessed via business information provider GENIOS (https://www.genios.de/). The data showed that two magazines had considerably greater financial resources than the other magazines, with the latter having annual budgets of far less than 1 million euros. To improve the comparability of the interviewed magazines, we excluded these two magazines and contacted the remaining *N* = 14 magazines, of which *N* = 7 agreed to be interviewed (three represented by women, four by men). All the magazines are based in Germany.

We sent the interview request via email using the contact addresses from the online magazines’ websites. We asked to be referred to a person involved in the online activities of the magazine. This resulted in interviews with mostly the editors-in-chief or publishers of their magazine that, in some cases, also were founding members. Due to the pseudonymization of the interviews as described in section “Qualitative content analysis,” neither the names of the online magazines nor the interviewees are disclosed, and paraphrases are used to make identification at least considerably difficult. We interviewed the following persons:

- Founder and coordinator of a platform with German news for non-German speakers (J01)
- Founding member and editor of a regional campus magazine (J02)
- Technical infrastructure manager of an online platform with minority perspectives on politics (J03)
- One of two managing partners at a local journalistic online magazine (J04)
- Founding member and editor-in-chief of a local weekly newspaper (J05)
- Founding member and editor-in-chief of an online magazine for uncovering fake news (J06)
- Editor-in-chief at an online magazine on digital topics (J07)

The interviews were conducted between July 7 and August 31, 2023. We made individual appointments for a Zoom call and recorded the interviews within the Zoom software. The interviews took 20–46 minutes, with an average interview time of 32:09 minutes (*SD *= 8.67). Before an interview began, each interviewee signed a written declaration of consent, which included the option to agree to the upload of pseudonymized transcripts to a research data management repository. After the interviews were conducted, a small gift was sent to the interviewees by post.

### Intervi19.448 ptew guideline

The interview questions were derived from the research questions shown above. After an introductory question, we addressed the overarching issue of visibility on the web. We asked about its importance, how it might be promoted, and what financial or human resources are available for online marketing measures. In the fourth part, we specifically focused on SEO, asking for reasons for and experiences of performing these measures. The interview was closed with a question on the interviewee’s general attitude towards SEO. The interview guideline is shown in Table 1.

**Table 1:** Interview guideline.

No	Question	RQ
Introduction		
0	At the beginning of the interview, please briefly tell us about your role within your online magazine.	
Visibility on the Web		
1	How would you describe the importance of visibility of your content on the web?	RQ1
2	Do you promote the visibility of your content on the web, and if so, how?	RQ2
Resources for promoting visibility on the Web		
3	How do you describe the resources your magazine has for online marketing activities?	RQ3
Search engine optimization*		
4.1	Why does your online magazine conduct SEO?	RQ2
4.2	Who carries out the SEO measures for your magazine (e.g., marketing agency, own staff)?	RQ3
4.3	What is your experience with SEO measures for your magazine?	RQ4
4.4	Concerning SEO measures in general: What attitude do you have as a representative of your online magazine on the possibility of improving the ranking of search results through SEO?	RQ5

* We also prepared questions for the event that an online magazine does not perform SEO. However, this was not the case with any of the magazines interviewed, which is why we have omitted the questions in the guideline.

### Qualitative content analysis

We prepared the data by transcribing the audio recordings and pseudonymizing the transcripts. Pseudonymization refers to substituting personal data with information that holds a similar meaning. This includes, but is not limited to, personal names, geographical locations, and company names. In the transcripts (see research data), the pseudonymized text segments were marked as follows: @@pseudonym##.

Using the software MAXQDA version 22.8.0, we then developed the main categories based on the interview guideline and, thus, the research questions. Next, we created the subcategories using a random subset of transcripts already coded with the main categories. This resulted in the coding scheme shown in the research data.

We coded the material in October 2023. We defined the unit of analysis as complete sentences to be assigned to a respective code. To determine the intra-rater reliability that indicates the stability of the coding process [58], a random sample of three transcripts was coded again after two months. We then calculated the agreement between both coding events using Cohen’s Kappa, resulting in a value of .906, *p* < .001. According to Landis & Koch [59], this strength of agreement can be regarded as “almost perfect.” The cases in which the two coding events did *not* match do not indicate any substantive discrepancies. For example, an interview statement on the importance of visibility was coded as “high for website” in the first and additionally as “high for general” in the second coding. Thus, since “high in general” was missing in the first coding, there is a discrepancy between both coding events that is reflected in Cohen’s Kappa.

## Results

### Gaining visibility on the Web

In this section, we look at the interview results for research questions one and two, that is, the importance of visibility on the web and measures to strengthen it.

As mentioned in the literature review section, non-profit magazines usually address topics rarely covered by larger commercial media organizations and pursue a societal mission. This is also reflected by the interviewees. For example, the non-profits’ articles are intended to educate people and prevent damage from others (J06), communicate German topics to non-German speakers (J01), and give room to independent voices for reporting on political issues (J03). To achieve these goals, the interviewees aim to establish their thematic priorities (J05) and reach as many readers as possible (J01, J03).

However, two considerations relativize the desire for visibility. They refer to the low level of competition and the absence of economic pressures. Due to the thematic unique selling points mentioned above, the representatives of the online magazines broadly identify no significant competition (J01, J02, J04, J06, J07). If similar offerings exist, these are not perceived as rivals but as valuable supplements to their content (J03). J04 describes the competitive situation as follows:

We are not in competition with anyone with our content. There are other players in the media landscape (...), [but] they do news. We don’t (...), but rather a more sustainable, slower form of journalism, (...) with longer research and more in-depth information (J04).

As a second limitation of visibility importance, the respondents emphasize that the survival of their non-profit magazines is not dependent on achieving certain traffic levels due to foundation funding and the absence of commercial interests (J01, J06, J07).

We are in a very privileged situation in that we are still completely funded by a foundation, at least at the moment. This means that even if things don’t perform very well (...), we’re still all right. (J01)

I mean, we don’t depend on that [traffic]. But there are colleagues out there whose business model is based on it. (J06)

We are in a relatively comfortable situation, not being dependent on commercial interests or on any commercial sponsors. (J07)

To achieve the described visibility on the web, online magazines engage in a range of online marketing measures. In addition to social media marketing, which all providers use via profiles on Facebook, Instagram, and sometimes X (Twitter), we below focus on the statements on search engine marketing activities PSM and SEO.

PSM plays a relatively minor role for the magazines. J02 and J04 occasionally placed Google Ads but no longer do so at the time of the interviews. J01 is considering using search engine ads in the future. Only J05 is currently running ads on Google, but only for a short period, so the effectiveness of the ads cannot yet be evaluated.

Compared to PSM, SEO is of higher priority as it is performed by all magazines, primarily driven by the high priority of search engine traffic. As J02 and J05 state, their magazines achieve a significantly higher traffic share via search engines than social media. J01 confirms this and adds that there has been a recent trend towards a decrease in traffic via social media. These observations led to search engine traffic becoming even more important than it used to be, resulting in the decision to strengthen the magazines’ focus on SEO. Besides the importance of traffic, there are also other reasons for the magazines to focus on SEO. J03 and J07 were influenced by their experiences from previous employers, which were for-profit magazines. SEO played a significant role for these companies, and the interviewees transferred this priority to their current positions at non-profit magazines. For J06, the relaunch of their magazine’s website was a decisive factor for starting SEO activities, as it was during this relaunch that the topic of SEO first came up for the editorial team. SEO continues to be part of the editorial process to this day to generate outreach for pieces that have been extensively researched and are intended to fulfill a societal mission.

We do research that is highly time-consuming and can take weeks. And we have an evidence-based approach and therefore (...) follow scientific standards. And, of course, it’s great when we actually discover something that benefits the general public, which happens quite often. After all, we want to share what we think is important. (J06)

In summary, the statements reveal a strong, albeit not economically decisive, desire for visibility on the web. This desire is primarily met through social media and SEO activities. In the next part of the results section, we will go into more detail on implementing SEO activities in the editorial offices.

### SEO as a shared task in everyday editorial work

In the following, we address the responses to research questions three and four, i.e., experiences and resource issues regarding SEO.

SEO measures require personnel or financial resources, which, according to the interviewees, lead to bottlenecks in other areas, such as text production.

[We are] primarily tied down by personnel capacities (...). And because we are a small editorial team and still try to somehow cover current topics and publish regularly, a large part of the team is involved in text production, translation, and editing so that something like that [SEO] tends to run “on top.” (J03)

It hurts when you are a nonprofit organization and think [...]: “I want to use the money to pay my authors and the photographer and not [...] this technology.” (J06)

Due to these constrictions, most magazines do not have the financial resources for continuous SEO support. Only J03 receives ongoing consultancy from an SEO agency, for which the interviewee is the contact person. An agency previously supported the online magazine of J06, but only temporarily as part of the website relaunch. For the others, SEO services are not affordable.

I literally fell off my chair at how expensive this [SEO agency service] is and the hourly rates they charge. That was completely impossible for us. (J05)

As a result of these limited personnel and financial resources, SEO is seen as a task to be tackled as a team and parallel to text production.

[SEO] is integrated into our daily routine – what works, works, and what doesn’t work, doesn’t work. I can (...) imagine that if we had someone doing it [SEO] full-time, we would end up somewhere else [higher in the search results ranking]. (J01)

The WordPress SEO plugin Yoast is usually used to support the editors (J01, J02, J04, J06). In addition, J02 and J06 provide their journalists with recommendations on the choice of title, use of keywords, headline formatting, and other SEO-relevant aspects.

The authors are (...) asked to make sure that they use the appropriate keywords to characterize the article. We also try to use (...) shorter formulations and link to other media. (J02)

[Placing the most important keywords for the articles] is something our content managers always have on their minds. (J03)

Despite the personnel efforts put into SEO, the interviewees noted only limited measurable success. Only J02 explicitly describes positive SEO experiences, namely that SEO measures have led to integrating their articles in Google News, resulting in a sharp increase in traffic. In contrast, J05 hoped for strong positive effects but is disappointed as these have not yet been achieved. The remaining respondents were either unable to assess the success of their SEO measures or were unsure whether perceived effects are attributable to SEO. For example, J01 and J06 noted an increase in traffic and suspect that this could have been due to their SEO efforts but also to other factors. For instance, J01 assumes that the shift in traffic share from social media to search engines might not (only) be due to successful SEO work but also to a decline in absolute social media traffic. J06 also noticed an increase in website traffic after a website relaunch and simultaneous implementation of SEO measures but is unsure which of these two factors was decisive for the increase in traffic.

To summarize, SEO is seen as a shared task in the editorial offices of nonprofit online magazines, as limited resources are available for agency support. Whether the self-conducted SEO efforts pay off in traffic growth remains largely unclear from the interviewees’ stance.

### Attitudes towards SEO

In this last result section, we examine the respondents’ attitudes towards SEO (RQ5), starting with the positive aspects of SEO. J01, J03, and J07 emphasize that from the user perspective, SEO is a valuable tool for making relevant content findable as long as it is carried out responsibly.

When used with a sense of proportion in such a way that we try to ensure that our content is found by the people who are interested in it [...], [SEO] is great. (J01)

After all, we are not only producers of online content but also consumers. And in that sense, we’re somehow dependent on [...] Google providing the content that actually interests us as readers. And that’s why I think it makes sense for website operators to think about this [SEO]. (J03)

In addition to these positive SEO effects, J05 and J06 take a pragmatic position on SEO by recognizing SEO as a matter of fact and a relevant topic, which it would be pointless to ignore.

It’s just the way it is. [...] You can’t get rid of it [SEO]. [...] It’s there and you have to make it work. (J06)

Apparently, people familiar with it [SEO] say it’s essential. And that you have to implement search engine optimization measures to ensure that everything runs smoothly. But I don’t know it. (J05)

Four interviewees explicitly mentioned negative aspects that would go hand in hand with SEO (J01, J02, J03, J04). A major risk is considered to be overly rigid keyword specifications for the editorial team, which could significantly interfere with journalistic freedom.

I also teach at the journalism school and sometimes hear what people from large media companies say: the head of department shows up in the morning with ten keywords they have to include in their articles by the end of the working day so that the articles are easily found. I’m very glad that I don’t work there [for large companies]. (J01)

The same aspect of strongly keyword-driven optimization is mentioned by J04, adding that this contradicts quality journalism.

I have a sense of apathy towards the exaggerated search engine optimization that is taking place in some companies, where you can speak of a “plague of digitalization.” In these media houses, large displays are hung in the newsrooms showing real-time click counts. That’s where I think quality journalism ends. (J04)

Also drawing a comparison with large media companies, J03 perceives a risk of displacement from financially stronger content providers who, unlike nonprofit providers, have resources available for SEO.

Media companies with extensive resources can simply outshine others. And that’s what I consider to be a problem. (J03)

As a further problem associated with SEO, J02 adds that over-optimized content offers little added value for users.

What I find unspeakable are [...] websites that [only] consist of SEO efforts and affiliate links, without any added value in terms of content. (J02)

In summary, interviewees have a nuanced approach to SEO, balancing pragmatic and positive views with critical tones that consider both their own and their readers’ interests.

## Discussion

Our interviews with representatives of non-profit online magazines demonstrated that, to them, visibility on the Web is not particularly important for economic reasons. Instead, these organizations aim for visibility to achieve societal goals, such as educating their readership about fake news or promoting minority perspectives on politics. To reach their full potential readership, search engine optimization (SEO) is considered an important tool, which all the magazines surveyed practice. This emphasis on SEO is similarly noted in the literature reviewed, particularly in interviews with journalists from more commercially oriented media organizations (e.g., [40,41]). However, as their resources are limited, non-profits must carry out SEO in parallel with other tasks. Furthermore, our respondents were uncertain whether the SEO measures they had implemented on their own were actually successful.

The representatives of non-profit magazines have a mixed attitude towards SEO. The interviewees were critical of SEO as it may potentially jeopardize journalistic freedom and quality, which is in line with other interviews with journalists [40,41,46–48]. However, the interviewees also positively recognize the potential of SEO to communicate content to their readership when used moderately, similar to the journalists and content providers interviewed by Schultheiß & Lewandowski [41] and Lopezosa et al. [40].

The interview results raise concerns about the visibility of quality journalism on the internet. The ubiquity of SEO creates pressure to adapt. Sufficient resources must be available for search engine marketing measures to generate traffic and subscriptions for for-profit organizations or increase the effectiveness of socially relevant content for non-profit organizations. Without these resources, achieving visibility on the Web is almost impossible.

In competing for peoples’ attention on the Web, for-profit providers have a significant advantage over non-profits. The latter are aware that they cannot compete for visibility with for-profit providers on popular topics due to their limited resources. Instead, they focus on niche topics, which allow them to reach a specific audience. Search results on popular topics are, therefore, dominated by for-profit offerings. Due to economic pressure, these offerings are subject to tendencies such as the so-called tabloidization of content, meaning a decline in quality due to adaptation to tabloid media with sensationalist news values and a popularized and personalized journalistic style [60]. Such tendencies are likely to be less pronounced for non-profit content. However, as users are predominantly confronted with profit-oriented content, they may miss out on the often higher-quality content of non-profit providers. As this situation can potentially lead to less well-informed citizens, the question of the responsibility of search engine providers in disseminating journalistic content to the public is raised.

Our study has limitations. Firstly, we only focused on online journalism and did not consider other non-profit sectors. Secondly, we interviewed a narrow target group of non-profit online magazines from Germany, which resulted in a relatively small sample size of seven interviews. This limited sample is not representative of all non-profit magazines in Germany, which restricts the generalizability of our findings. Although the interview responses provided a coherent overall picture, it is unclear whether we achieved data saturation during the sampling process [61]. Therefore, we cannot be certain if different perspectives would have emerged had we included a broader range of non-profit online magazines. Lastly, we recognize that interviewer effects may have influenced the responses of our interviewees. However, since the interviews were consistently conducted by the same interviewer and based on a structured questionnaire, we believe that potential biases from interviewer effects have been minimized.

To our knowledge, this interview paper is the first to explore the area of non-profit journalism in relation to search engine visibility. Non-profit journalism plays a vital role in society, covering areas such as investigative reporting, health topics, and social justice issues, yet it often receives limited visibility in search engines. Therefore, this paper serves as a starting point for further research in this important context.

The limitations of this study can provide direction for future research. First, our study contributes to gaining insights into the perspective of non-profit providers in the competition for users’ attention on the Web. Future research can build on that and address how non-profit journalists perceive the role of search engine providers in disseminating journalistic content to the public. Secondly, it must be noted that non-profit journalism, which was the focus of our study, is just one example of many non-profit sectors that are likely to face similar challenges. These sectors also compete with larger organizations that have greater resources, such as state vs private educational institutions, state vs private clinics, and environmental organizations vs large corporations. Therefore, we deem it necessary to conduct further research to obtain additional perspectives from other non-profit sectors and countries.

## Conclusion

In this article, we have analyzed the viewpoints of non-profit online magazines concerning their online presence. We have conducted semi-structured interviews with representatives of non-profit online magazines in Germany and inquired about the significance of online visibility for them. Additionally, we have asked how they manage to achieve visibility to reach their target audience, especially considering their limited resources. We specifically focused on search engine optimization (SEO), a set of methods to increase the ranking of web pages in search engines.

The interview findings revealed that search engine visibility is crucial for non-profit organizations to achieve their societal goals, rather than for economic reasons. These goals include educating their audience about fake news or promoting minority perspectives on politics. All magazines surveyed considered SEO an important tool to reach the maximum number of people possible. However, due to limited resources, performing search engine optimization measures is only a secondary task and cannot receive significant effort as it diverts attention from primary tasks such as content production.

The study enhances our understanding of how non-profit magazines perceive the challenge of establishing their online presence when compared to their for-profit counterparts with significantly larger marketing budgets. This leads us to the question of how both for-profit and non-profit quality journalism can be distributed fairly to the public. Future research should explore this question and evaluate what role search engine providers (see, for example, the Google News Initiative, https://blog.google/outreach-initiatives/google-news-initiative/) could play in assisting non-profit organizations in reaching their desired audiences. Additionally, it is important for future studies to focus on the rapid advancements in artificial intelligence (AI). For instance, the integration of AI-generated summaries in search engine results, like those on Google or Bing, could introduce new opportunities or risks for the visibility of non-profit journalism. Furthermore, sectors beyond non-profit journalism and their perspectives on visibility on the Web should be further investigated.
